# Improved Veterinary Service, United States Army

**Published:** 1901-03

**Authors:** D. E. Salmon, Rush S. Huidekoper, Leonard Pearson, A. W. Clement, Wm. Herbert. Lowe

**Affiliations:** Washington D.C.; Washington D.C.; Washington D.C.; Washington D.C.; Washington D.C.


### SOCIETY PROCEEDINGS.

### IMPROVED VETERINARY SERVICE, UNITED STATES ARMY.

(Concluded from page 124.)

An analysis of the arguments which had been used before the Committees, and in both Senate and House of Representatives, shows that the only one with seeming reason is that of the Secretary of War, that he desires to diminish the staff corps and does not want to add an additional one. A prejudice was engendered that the rank of colonel was too high for such an organization.

Many of the Senators assumed that "rank" was given in the Senate amendment, section 16, and the majority of Senators conceded that they approved of giving the rank of second lieutenant to the veterinarians of the cavalry and artillery.

On Friday, December 11th, our Secretary was given an appointment by the Secretary of War and submitted to him two paragraphs which would remove the objections above, requesting his consideration of them and approval for an amendment.

These were:

That the veterinarians provided for in section 16 of this act for regiments of cavalry and artillery shall have the rank, pay, and allowances of a second lieutenant, mounted.

And:

That there shall be one chief veterinarian, with the rank, pay, and allowances of a major, attached to the Quartermaster-General's Department.

The Secretary of War said that he would approve the first. He said that he would refer the other to the Quartermaster-General; to return next day for an answer.

[28] Our Secretary returned to the War Department several times without being able to see the Secretary of War. Finally, on Tuesday afternoon, in response to his card, a clerk brought the paper with the second paragraph blue lined through and the statement "the Secretary says he disapproves this (indicating the blue pencilled), but has no objection to the other. He is busy and will see no one." A request for the approval promised for the paragraph for rank for the second lieutenants was met by a repetition of the answer and a refusal to return to the Secretary with a card. As our Secretary represented our committee and an organizaton of several thousand professional men and American citizens, we deem that we should have had the courtesy of a direct answer from the Secretary of War.

These paragraphs were not referred to the Quartermaster-General.

Senator Gallinger then offered an amendment covering this proposition.

This was acted upon on the day of the passage of the bill.

The following extracts are from the *Congressional Record* of January:

Mr. Gallinger: Mr. President, I offered two amendments to this bill, which at the proper time will be reached for consideration, and I desire to occupy a very few minutes in explanation of those amendments, it probably being the only opportunity I shall have to do so.

It will be remembered that during the debate on the veterinary clause of this bill quite a number of Senators—and I think I am safe in saying some of them belonging to the Committee on Military Affairs—said that they had no objection to giving proper rank to these educated men, and as that recommends itself very warmly to my view, I propose to offer an amendment which will give the ordinary veterinarian what I think is proper and adequate rank in the army.

The amendments that I propose are to section 16, the first one being to insert simply the words "with rank," so as to read: "Shall have the rank, pay, and allowances of second lieutenants, mounted."

In addition to that, Mr. President, I intend to offer an amendment, to which there may be very likely more objection, possibly, than to the first one I have named, in these words:

"And there shall be a chief veterinarian, with the rank, pay, and allowance of a major, who shall be attached to the Quartermaster-General's Department."

Mr. President, I believe that the rank of second lieutenant ought to be accorded to all of those skilled men, men of high education, without objection on the part of the Senate. I feel that the Committee on Military Affairs of the Senate ought at least to permit this matter to go into conference and give it more mature and careful consideration than can be done in debate in the Senate.

As to the second amendment, which proposes a chief veterinarian, with the rank of major—not a very high rank—I am equally of the opinion that that ought to be allowed to go into the bill.

When this matter was under discussion before, the senior Senator from Massachusetts [Mr. Hoar] suggested that, in his opinion, there ought to be a college in some way attached to the army whereby men could be educated for the position of veterinarian. Why, Mr. President, there are colleges in this country devoted to this purpose, and it is a well-known fact to the profession that every branch of medical study that is required in the medical colleges and universities of the United States is required in those colleges. Their graduates have to pass in surgery, in the practice of medicine, in therapeutics, and in all the other branches, with one or two exceptions, that are taught in the ordinary medical colleges.

In addition to that, they have special courses in reference to the diseases of dumb animals, and I will venture to say here that there is very little difference between the diseases that afflict the man and those that afflict the horse. They are substantially of the same kind, and the same knowledge that enables a man to treat a dumb animal enables him to treat a man, *vice versa*.

But, Mr. President, what I want to emphasize more particularly was that the men who graduate from the veterinary colleges of the United States at the present time are among the highest educated medical men there are in this country.

It does seem to me that this important branch of the service ought at least to have one man at the head of it with adequate rank—and I propose that of major—who would be connected with the War Department and who would have under his immediate supervision the important matters relating to the cavalry branch of the military service of the United States. That is all I care to say about that.

Appealing, as I will in closing, to the Committee on Military Affairs to permit these amendments to go into the bill and then to give them careful and considerate attention when they are before the Committee of Conference—

Mr. Sewell: Will the Senator allow me a word?

Mr. Gallinger: Certainly.

Mr. Sewell: As the bill stands, the question will be open in conference without any further amendment being now made.

Mr. Gallinger: I am not quite so sure about that, but it certainly will not be before the Committee of Conference in the form I propose; and I appeal to the Senator that it will do no harm to let this matter go into conference in the shape in which I have placed it. I shall certainly find no fault with the conclusion which the Conference Committee may reach, whether it agrees with my view or not.

. . . . . . . .

Mr. Gallinger: I submit the amendment which I send to the desk.

The President *pro tempore*: The amendment will be stated.

The Secretary: On page 30 amend section 16 by striking out in line 2 the words "receive the" and by substituting the words "have the rank," and at the end of the section add the words, "and there shall be a chief veterinarian, with the rank, pay, and allowances of a major, who shall be attached to the Quartermaster-General's Department," so that the section, if amended as proposed, will read:

[29] "Sec. 16. That the grade of veterinarian of the second class in cavalry regiments, United States Army, is hereby abolished, and hereafter the two veterinarians authorized for each cavalry regiment and the one veterinarian authorized for each artillery regiment shall have the rank, pay, and allowances of second lieutenants, mounted. Such number of veterinarians as the Secretary of War may authorize shall be employed to attend animals pertaining to the Quartermaster's or other departments not directly connected with the cavalry and artillery regiments, at a compensation not exceeding $100 per month; and there shall be a chief veterinarian, with the rank, pay, and allowances of a major, who shall be attached to the Quartermaster-General's Department."

Mr. Gallinger: I ask that the proposed amendment be divided. It relates to two entirely different subjects.

The President *pro tempore*: The question is on agreeing to the first amendment offered by the Senator from New Hampshire. [Putting the question.] The noes seem to have it.

Mr. Pettigrew: On that I ask for the yeas and nays.

The yeas and nays were not ordered.

The amendment was rejected.

Mr. Gallinger: Let the question be taken on the second amendment.

The President *pro tempore*: The Senator from New Hampshire offers an amendment to the last part of the section, which will be stated.

The Secretary: Add to the end of section 16, page 30, the following:

"And there shall be a chief veterinarian, with the rank, pay, and allowances of a major, who shall be attached to the Quartermaster-General's Department."

M. Gallinger: I will withdraw that amendment, the former amendment having been rejected.

Later, Senator McCumber offered an amendment as follows:

Mr. McCumber: I offer the amendment I send to the desk.

The Presiding Officer: The Senator from North Dakota offers an amendment which will be stated.

The Secretary: After the word "mounted," in line 3, page 30, section 16, it is proposed to insert the following words:

"And the sum of $1800 per annum after five years' service."

The Presiding Officer: The question is on agreeing to the amendment offered by the Senator from North Dakota.

Mr. Kenney: Let it be again stated.

The amendment was again stated.

Mr. Kenney: May we ask what print the Secretary is reading from. Is it the print of January 10?

The President *pro tempore*: It is. The paragraph as proposed to be amended will be read.

The Secretary read as follows:

"That the grade of veterinarian of the second class in cavalry regiments, United States Army, is hereby abolished, and hereafter the two veterinarians authorized for each cavalry regiment and the one veterinarian authorized for each artillery regiment shall receive the pay and allowances of second lieutenants, mounted, and the sum of $1800 per annum after five years' service."

The amendment was rejected.

### Summary.

From a legal aspect, the Veterinary Corps section of the Army bill, the amendment offered by Senator Kenney to the Army Efficiency bill, S. 4300, passed the Senate May 4, 1900, opposed by the Committee on Military Affairs of the Senate; passed the House of Representatives December 6, 1900, opposed by the Committee on Military Affairs of the House; and then, with the title and enacting clause, constituted the original bill enacted by the majesty of a majority of both Houses, beyond any action of a Conference Committee and awaiting only the signature of the President. To this were added amendments by the House.

By some fiction of procedure, when this bill, S. 4300, was *returned* to the Senate, it was referred to the Committee on Military Affairs, and on reaching the Senate the Veterinary Corps section was taken up for reconsideration, not within the customary forty-eight hours, but after eight months.

It was nevertheless reconsidered by the Senate, acted upon a second time, and replaced by an amendment.

The one argument, with any seeming reason, which has been advanced in opposition to the Veterinary Corps, is that of the Secretary of War, who desires the diminution of the number of staff corps and Bureau of the War Department, and who objected to the establishment of a new one. As a general proposition this appeared to have some logic, and was insisted upon, in a number of speeches, in the debates on the floor of the Senate and House.

While this objection to the staff corps may be expedient and proper in regard to the supply departments, it is not in any way applicable to the Veterinary Corps.

The veterinary service is, like the medical service, intended to meet certain specific technical ends which require specially adapted professional men. The officers of neither the Medical Corps nor the Veterinary Corps can be made interchangeable with those of other corps or with the "line," as it is suggested that the officers of the more strictly military corps, ordnance, quartermaster, commissary, etc., can be.

A Veterinary Corps, properly established, with "rules and regulations" . . . "made by the Secretary of War," in conformity with the customs of the United States service, could in no way interfere with or complicate the fixing of responsibility or interfere with the authority of commanding officers.

The following from the Regulations of the Veterinary Service in France (*Bulletin Official du Ministère de la Guerre; Service Vétérinaire de l'Armée*) gives the general outline of the functions of the service.

[30] CHAPTER I. OBJECT OF VETERINARY SERVICE. "ART. I. The veterinary service of the army has for its object the preservation of the health of its animals; the treatment of those afflicted with disease; farriery and horseshoeing; the inspection of animals for butchery, and the examination of meat intended for food in garrison and in campaign.

"ART. 2. The veterinary service is furnished in peace (*a l'interieur*) by a special force of military veterinarians; in war (*en campagne*) by this same corps, augmented by the reserve and territorial veterinarians.

"ART. 3. The veterinary service acts under military authority and is always subordinate to it. Its organization is defined by special instructions of the minister of war. These regulations go on to define the location of the veterinarians; in time of peace.

"The superior officers to headquarters, army corps, and divisions, whose duties are those of inspectors of contagious diseases, hygienic conditions, annual inspections of stables, horses, etc.

"Those of the next grades as instructors of the cavalry schools and the schools of farriery.

"Those of the lower grades to practical services with the mounted troops.

"In time of war: The disposition of veterinarians necessitated by military operations and local conditions is prescribed by the generals commanding the army and army corps.

"The chief having direction of the supplies (medicines, instruments, and dressings) for the service is charged with inspection of its personnel and their duties and with their reports, for the information of the head of the army."

Further duties prescribed are: Inspection and chemical analysis of forage; inspection of shoeing and feet when shod; autopsy of animals which die; examination of horses for purchasing boards; inspection of hygienic conditions in transport; disinfection after contagious diseases; details of meat inspection; reports through military channels, etc.

The rules and regulations of the veterinary service in England, Germany, Austria, Belgium, and Italy differ little from those of France.

A great deal has been said against the rank of colonel for the chief of the Veterinary Corps.

In England and most European countries the chief of the veterinary service has the rank of colonel. Belgium gives only a lieutenant-colonelcy. In Russia the veterinarian attains the rank of general.

As we have seen in the outline of duties in the French service, which is similar to that of other countries, the chief veterinarian, in addition to hit bureau duties, acts as an inspector-general for a special line of his duties.

The same argument which obtains in our Inspector-General's Department, that the Assistant Inspector-Generals should have the rank of colonel, on account of their peculiar relations with the commanding officers of regiments, holds for the chief veterinarian having the same rank.

The Department of Agriculture pays its chief veterinarian $4000 a year —$500 more than a colonel's pay.

Five assistant veterinarians, $2500 a year—a major's pay.

A number $2250 and $2000 a year—a captain's pay or more.

The mass are graded at $1800, $1600, $1400, to which they are advanced shortly after commencing service at $1200 a year.

The State of Wyoming paid $4000 to its State veterinarian—$500 more than a colonel's pay. Montana pays $3000—a lieutenant-colonel's pay. New York pays the same to two veterinarians. Pennsylvania pays $2500 and all expenses—better than a major's pay, etc. These veterinarians are allowed private practice in addition.

If the State governments and United States government deem it wise to pay these salaries for competent veterinarians, the latter surely is warranted in giving equivalent pay to officers in charge of the veterinary service of the army.

Ignorant arguments were used in debate in both the Senate and the House against according "rank" at all to veterinarians.

When such aristocratic countries as Germany, who only accorded "rank" to her medical officers in 1847, and Russia give rank to the veterinarian it is absurd for a democratic country, in which class distinction is not supposed to prevail, to question the right of men to hold rank who belong to a profession which is honored by having such men in it as Simpson and Colonel Fleming, former Director-General of the veterinary service in the English army, both knighted by the Queen; Bouley and Chauveau, in France, both members of the French Academy; the Marquis Ercolani, of Italy; Renault, Delafond, Colin, Hertwig, Spinola, Gerlach, Ostertag, Friedberger, Nocard, Schuetz, and numerous men of scientific standing equal to that of any medical men. Pasteur and Koch had for their most active co-laborers veterinarians of world-wide reputation.

That some system is required in our army is evidenced by the fact that the civilian veterinarians now employed send in, through their quartermasters, heterogeneous requisitions so dissimilar that the Quartermaster-General had asked an expert to classify and tabulate them into some practical shape.

We believe that there is no knowledge in the War Department to-day of how these supplies are expended or of what the majority of animals die, whether preventable or not.

We have been criticised as holding to and insisting upon the corps formation and too high rank.

We adopted a draft of an organization found practicable and efficient by all other countries. We submitted it to the Secretary of War in December, 1899, asking for action upon it, and seeking such modification and alteration as would make an efficient service and be acceptable to the War Department.

The Secretary of War said that he would refer it to General commanding and to the Quartermaster-General. He did not.

In January, 1900, he said he would not consider the proposition. We were, therefore, obliged to persevere in the line established, and during the last year or more over 3000 veterinarians, assisted more or less by an immense number of the other 5000 veterinarians in the country, have taken an active interest in this legislation, because we believed that now, when the army was to be reorganized under a bill whose title indicated that *efficiency* was the object, was the proper time for favorable consideration.

The Senate passed the Veterinary Corps section.

In November, 1899, we wrote to the Adjutant-General, still hoping for consideration and such modification as would be acceptable to the Secretary of War. The Adjutant-General did not answer our letter.

The House passed the Corps section in the same bill in which it had passed the Senate.

After the adverse action of the Senate on January 7th we submitted to the Secretary of War two paragraphs, providing for the rank of second lieutenant for cavalry and artillery veterinarians and an officer, with the rank of major, in the Quartermaster-General's Department.

The Secretary of War said that he would approve the first and would refer the second to the Quartermaster-General. Four days later he returned the first paragraph by a clerk, with a verbal message that he had no objection to it, and objected to the other. He did not refer it to the Quartermaster-General.

Sentor Gallinger offered these paragraphs as an amendment to the Army bill in the Senate. They were rejected.

We will at a later time render another report and suggest an outline for future action.

Respectfully submitted,

[Signed] D. E. SALMON,
RUSH S. HUIDEKOPER,
LEONARD PEARSON,
A. W. CLEMENT,
WM. HERBERT LOWE.

WASHINGTON, D. C., January 23, 1901.

*To Dr. Tait Butler, President, and the Executive Committee of the American Veterinary Medical Association.*

